# Editorial: Actinomycete natural products: isolation, structure elucidation, biological activity, biosynthesis, and yield improvement

**DOI:** 10.3389/fchem.2024.1471029

**Published:** 2024-08-09

**Authors:** Susana P. Gaudêncio, Wasu Pathom-aree

**Affiliations:** ^1^ Associate Laboratory i4HB, Institute for Health and Bioeconomy, NOVA Faculty of Sciences and Technology, NOVA University of Lisbon, Caparica, Portugal; ^2^ UCIBIO, Applied Molecular Biosciences Unit, Chemistry Department, NOVA Faculty of Sciences and Technology, NOVA University of Lisbon, Caparica, Portugal; ^3^ Department of Biology, Faculty of Science, Chiang Mai University, Chiang Mai, Thailand

**Keywords:** actinobacteria, natural products, isolation and structure elucidation, drug discovery, biotechnological applications, blue biotechnology

## Introduction

Natural products from Actinobacteria,Hsi commonly known as actinomycetes, have historically provided humans with numerous antibiotics (e.g., streptomycin, gentamicin, and vancomycin) (Schatz et al., 1944; Cooper and Yudis, 1967; Rake et al., 1986), anticancer agents (e.g., doxorubicin, bleomycin, and calicheamicins) (Shastri et al., 1971; Maiese et al., 1989), and agrochemicals (e.g., avermectin and spinosad) (West, 1996; Molinari et al., 2010). It should be highlighted that approximately two-thirds of all approved antibiotics are derived from actinomycetes, predominantly by *Streptomyces* species, underscoring the importance of these microorganisms (Barka et al., 2016).

The discovery and biological evaluation of new natural products from actinomycetes is an endless frontier in the post-genomic era, primarily driven by advances in microbial genomics and synthetic biology. Understanding the biosynthesis of actinomycete natural products not only elucidates how nature constructs these complex molecules from small building blocks, such as amino acids and acyl-CoA, but also provides a basis for improving their yields for industrial development. Some natural products feature unprecedented structural scaffolds and impressive biological activities, inspiring synthetic and medicinal chemists to design and synthesize the next-generation of medicines. Additionally, actinomycetes have the advantage of enabling the retrieval of natural products through fermentation techniques.

The Research Topic “Actinomycete natural products: isolation, structure elucidation, biological activity, biosynthesis, and yield improvement” includes five articles reporting original research. The research contributions span from exploring diverse actinomycete origins, such as ocean sediments and atmospheric-derived actinomycetes, to the characterization and identification of actinomycete isolates and the dereplication, isolation, structural elucidation and configuration determination, bioactivity testing and toxicity evaluation of novel secondary metabolites, as well as biosynthetic pathway analysis. The five contributions are described below by publication date.

## 2 Natural products, including a new caboxamycin, from *streptomyces* and other actinobacteria isolated in Spain from storm clouds transported by northern winds of arctic origin

Atmospheric-derived actinomycetes represent a novel and promising source for drug discovery, traditionally focused on terrestrial and marine environments. The isolation of 18 bioactive actinomycete strains from storm clouds over Northern Spain highlights an innovative perspective on their natural habitat and dispersal. Air masses traced to the Arctic Ocean, Canada, Greenland, and Europe suggest that atmospheric pathways significantly influence microbial distribution and diversity. Detection of 94 secondary metabolites, including 69 known and 25 novel compounds, underscores the chemical diversity and potential biological activities of these microorganisms. The pioneering isolation and structural determination of caboxamycin B from *Streptomyces* sp. A-177 marks the first novel natural product from an atmospheric *Streptomyces*. This study emphasizes the importance of bioprospecting atmospheric environments to discover new bioactive compounds with significant pharmaceutical applications, broadening our understanding of microbial ecology and enhancing prospects for innovative drug development.

## 3 Crystal structure of the α-ketoglutarate-dependent non-heme iron oxygenase CmnC in capreomycin biosynthesis and its engineering to catalyze hydroxylation of the substrate enantiomer

The study delves into α-KG-dependent non-heme iron oxygenases, focusing on CmnC in antibiotic biosynthesis. Comparing CmnC with VioC and OrfP reveals conservation in substrate binding despite differing substrate specificities, particularly in hydroxylating d-Arg. Identification of critical residues (Leu136, Ser138, Asp249) in CmnC, distinct from OrfP, enhances understanding of enzyme-substrate interactions. Engineering CmnC to favor d-Arg with the triple mutant CmnCL136Q, S138G, D249Y showcases potential for tailored enzyme activity via mutagenesis. These findings extend to biocatalysis and drug development, facilitating the design of novel biocatalysts for chiral compound synthesis and antibiotic production. Insights into biosynthetic enzyme nuances like CmnC also inform strategies for discovering and optimizing therapeutic agents.

## 4 An in-cluster Sfp-type phosphopantetheinyl transferase instead of the holo-ACP synthase activates the granaticin biosynthesis under natural physiological conditions

This study enhances understanding of polyketide biosynthesis, focusing on acyl carrier protein (ACP) activation by phosphopantetheinyl transferases (PPTases). Gra-ORF32 in *Streptomyces vietnamensis* is identified as a dedicated PPTase for granaticin biosynthesis, featuring a crucial Arg- and Pro-rich N terminus that underscores its functional separation from fatty acid biosynthesis. Various endogenous and exogenous PPTases demonstrate broad substrate recognition, albeit with differing efficiencies, highlighting the enzymes’ evolutionary flexibility. Crosstalk between the granaticin and kinamycin-like pathways on ISP2 plates illustrates pathway complexity and potential for enhancing secondary metabolite production through engineering. The strict regulation of host FAS ACPS and its inability to replace Gra-ORF32 under natural conditions reveals precise control mechanisms in polyketide biosynthesis, crucial for type II PKS reconstitution in heterologous hosts like *E. coli*. This research contributes significantly to natural product biosynthesis and metabolic engineering by elucidating PPTase roles and regulatory mechanisms, offering insights for pathway optimization and the development of new antibiotics and therapeutics amid increasing antimicrobial resistance.

## 5 Nocaviogua A and B: two lipolanthines from root-nodule-associated *Nocardia* sp.

The discovery of Nocaviogua A and B expands the known diversity of natural products, particularly within the emerging class of lipolanthines. These compounds, featuring a non-canonical avionin (Avi)-containing macrocycle and a long acyl chain, were isolated from a mutualistic actinomycete associated with sea buckthorn root nodules in Tibet. This context highlights the importance of exploring unique ecological niches for novel bioactive compounds. The structural elucidation of Nocaviogua A and B, including their absolute configurations, marks the first comprehensive characterization of this family of lipolanthines. This finding adds to the chemical diversity known from natural sources and provides a basis for future chemical and biological studies. Although Nocaviogua A exhibited only weak cytotoxicity against the tested cancer cell lines, this initial assessment is crucial for understanding the biological potential of lipolanthines.

## 6 Novel metabolite madeirone and neomarinone extracted from *Streptomyces aculeoletus* as marine antibiofilm and antifouling agents

Madeirone and neomarinone, identified from *Streptomyces aculeoletus*, offer promising solutions to marine biofouling, a pervasive challenge affecting industries such as shipping and aquaculture. With the global ban on organotin compounds used as antifouling agents, there is a critical need for environmentally friendly alternatives. These compounds exhibit potent antibiofilm activity against marine fouling bacteria and effectively prevent the settlement of marine larvae without harming bacterial viability. *In silico* toxicity predictions and *in vivo* ecotoxicity studies on marine organisms support their potential as safe antifouling agents. The study also suggests avenues for exploring their biosynthetic pathways, modes of action, and potential modifications to enhance efficacy and safety. Furthermore, this research underscores the importance of investigating marine microorganisms for development of novel antifouling strategies, contributing to sustainable solutions for biofouling management.
